# Spatio-Temporal Change of Land Use/Land Cover and Vegetation Using Multi-MODIS Satellite Data, Western Ethiopia

**DOI:** 10.1155/2023/7454137

**Published:** 2023-10-31

**Authors:** Tesema Kebede Seifu, Tekalegn Ayele Woldesenbet, Taye Alemayehu, Tenalem Ayenew

**Affiliations:** ^1^Haramaya Institute of Technology, Haramaya University, P.O. Box 138, Dire Dawa, Ethiopia; ^2^Ethiopian Institute of Water Resources, Addis Ababa University, P.O. Box 1176, Addis Ababa, Ethiopia; ^3^School of Earth Sciences, Addis Ababa University, P.O. Box 1176, Addis Ababa, Ethiopia

## Abstract

Land use and land cover (LULC) change and variability are some of the challenges to present-day water resource management. The purpose of this study was to determine LULC and Normalized Difference Vegetation Index (NDVI) fluctuations in western Ethiopia during the last 20 years. The first part of the study used MODIS LULC data for the change analysis, change detection, and spatial and temporal coverage in the study region. In the second part, the study analyzes the NDVI change and its spatial and temporal coverage. In this study, The Moderate Resolution Imaging Spectroradiometer (MODIS) satellite data were applied to determine LULC and NDVI changes over four different periods. Evergreen broadleaf forests, deciduous broadleaf forests, mixed forests, woody savannas, savannas, grasslands, permanent wetlands, croplands, urban and built-up lands, and water bodies are the LULC in the period of analysis. The overall classification accuracy for the classified image from 2001 to 2020 was 85.4% and the overall kappa statistic was 81.2%. The results indicate a substantial increase in woody savannas, deciduous broadleaf, grasslands, permanent wetlands, and mixed forest areas by 119.6%, 57.7% 45.2%, 37%, and 21.3%, respectively, followed by reductions in croplands, water bodies, savannas, and evergreen broadleaf forest by 90.1%, 19.8%, 13.2%, and 4.8%, respectively, for the catchment between 2001 and 2020. The result also showed that the area's vegetation cover increased by 64% from 2001 to 2022. This study could provide valuable information for water resource and environmental management as well as policy and decision-making.

## 1. Introduction

Land use and land cover (LULC) change is a key driver of environmental change and is becoming a global concern due to its impact on the local, regional, and global environment [1–5]. Understanding how LULC classes affect upcoming precipitation as well as surface dynamics which are ecologically relevant is crucial for sustainable water resource management and ecosystem [6, 7]. LULC change is exacerbated by human activities such as population growth, urbanization, and deforestation [8]. Several studies have identified these changes as a current challenge to the ecohydrological system of the environment [8–12].

There is presently a wide spectrum of studies on LULC, on the database, taking place throughout the world utilizing various technological applications [13–18]. Remote sensing instruments are currently a widely used technology for analyzing LULC change in different regions of the world [19–21]. Landsat, Sentinel-2, MODIS, the main product of wide coverage, and high-repeatability remote sensing imagery were recently used for LULC and water resource applications [7, 22, 23].

NDVI (Normalized Difference Vegetation Index), the most common Vegetation Index among remote sensing techniques, can indicate the growth status, type, and biomass of vegetation and has a linear correlation with vegetation cover. NDVI is a vegetation indicator that is considered the best indicator of vegetation growth and cover [24–26]. Currently, scientists have obtained NDVI data from various satellites such as Landsat, Sentinel-2, and MODIS. Vegetation Cover Index analyzes were not conducted in the study area showing changes in NDVI in the area, despite some LULC studies showing slight LULC changes.

The remote-sensing images used in this article are from the USGS Earth Explorer and NASA websites. The Moderate Resolution Imaging Spectroradiometer (MODIS) is a satellite that collects remote sensing data used by scientists to monitor, model, and assess the effects of natural processes and human actions on the Earth's surface. MODIS has two sensors orbiting the Earth: the Terra (EOS AM) satellite, launched by NASA in 1999, and the Aqua (EOS PM) satellite, launched in 2002 [27–29].

Change in LULC is the major challenge in Ethiopia's hydroecological studies particularly in the study region [30]. The anthropogenic factor is the main source and results in a significant change in the LULC in Ethiopian watersheds. Key anthropogenic activities, including tree felling, land conversion to agriculture, and human settlement, are the major causes of LULC change. These changes in LULC were also recently studied by several researchers in Ethiopia [31–34]. Some researchers tried to study the LULC change in the study region [30, 35, 36]. However, the spatial scope of these works is limited and the period is also very short. Also, the methods used in previous studies do not address finished products for remote sensing applications such as MODIS. None of the researchers have yet dealt with the LULC change of western catchments considering the spatiotemporal patterns of LULC. The region considered in this particular study is an area ecologically rich and sensitive to climate and LULC changes. The novelty of this study consists in comparing the methods (LULC and NDVI techniques) and their spatial and temporal coverage of the study region. In addition, the study updates LULC and vegetation information by mapping changes in magnitude, trend, and spatial distribution. Identifying and mapping the LULC change of a given area are critical for understanding the vulnerable area creating a sustainable ecosystem for hydroclimatic management. Therefore, this study aimed to analyze the trends and changes in LULC and vegetation dynamics in western Ethiopia with MODIS satellite imagery between 2001 and 2020. The results of this study could provide firsthand information on the trend, extent and magnitude of LULC, and Vegetation Index in the study region. Furthermore, the study provides foundational information for LULC and vegetation-related information for regional and national governments, policymakers, stakeholders, and the local community.

## 2. Materials and Methods

### 2.1. Study Area Description

The study area is located in western Ethiopia and includes the Gambela Regional State in the west and some areas in the Oromia Regional State in the east. The area is part of the Baro-Akobo River Basin, which is one of the tributaries of the Nile River system. The catchment is located at latitudes 6°50′ and 8°43′ north and longitudes 33°00′ and 35°51′ east and covers an area of over 40889 km^2^. The area receives the highest amount of mean annual precipitation (1600 mm) and humidity is the climatic character of the region. The relative humidity in the region ranges from 62.8 to 77.9% while the mean temperature ranges from 18.08 to 28.8°C. The area is characterized by more than five rainy months between March and October. Forest and savanna are the most important LULCs dominating more than 70% of the area.

### 2.2. Data Acquisitions

**Figure 1:**
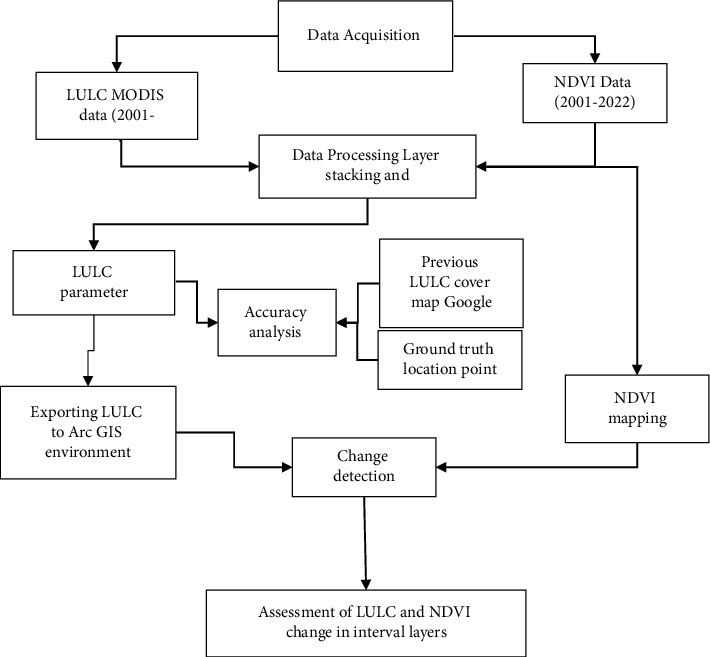
Framework of the study.

Two files were obtained and evaluated for this study: MCD12Q1 Land Cover Type (Collection 5) and MOD13Q1 Vegetation Indices. MODIS products were accessed from freely available data from the Earth Observing System Data and Information System (EOSDIS) Internet page. The MODIS Land Cover Type product includes many categorization systems that describe land cover attributes generated from observations spanning a year's worth of Terra and Aqua data input. We used the most current Collection 5 MODIS Global Land Cover Type product, which was released in 2020 and spans the years 2001–2020 with a spatial resolution of 500 m. For the years 2001–2022, the MODIS 16-day composite vegetation index product was examined. It has a spatial resolution of 250 m and comprises the Enhanced Vegetation Index (EVI) and the Normalized Difference Vegetation Index (NDVI). The overall method of procedure of the study is presented in Figure 1.

**Figure 2:**
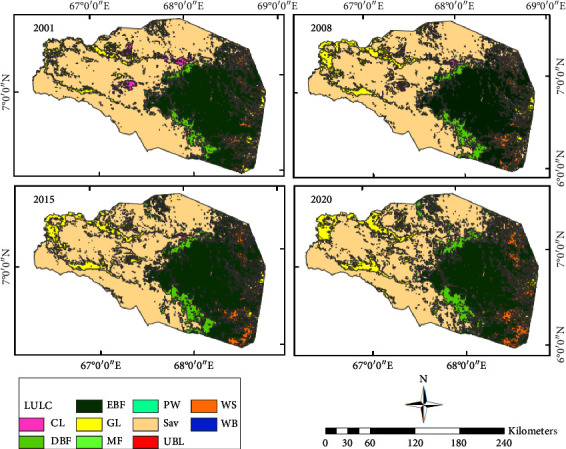
LULC change between 2001 and 2020.

**Figure 3:**
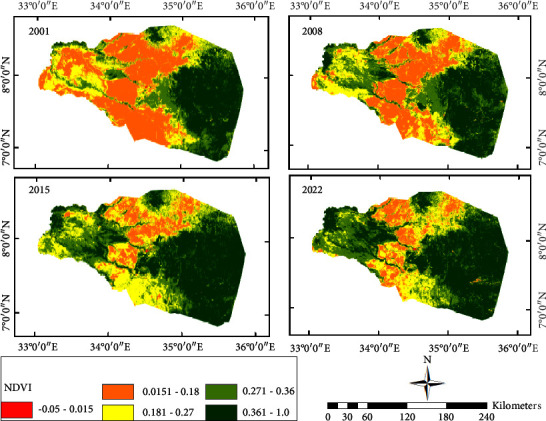
Spatial distribution of NDVI between 2001 and 2022.

**Figure 4:**
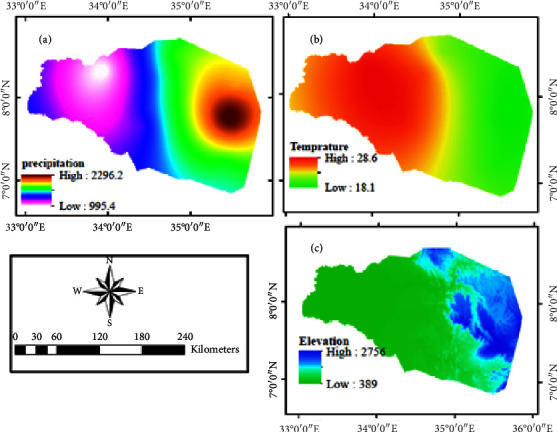
The climatic and topographic map of the study area.

LULC data (Figure 2), NDVI data (Figure 3), climate data (Figures 4(a) and 4(b)), and topographic data (Figure 4(c)) were used for this particular study. The Moderate Resolution Imaging Spectroradiometer (MODIS) product was chosen to analyze the spatial and temporal trends of LULC and NDVI. The MODIS LULC data from 2000 to 2020 were downloaded from the United States Geological Survey (USGS). For NDVI data, the MOD13Q1 MODIS was used from the NASA website (Table 1). MODIS satellite was launched and developed by NASA in 1999. MODIS is a key instrument onboard the Terra (originally known as EOS AM-1) and Aqua (originally known as EOS PM-1) satellites [27, 28]. For 2001, present, the MODIS Land Cover Type product (MCD12Q1) provides worldwide maps of land cover at yearly time steps and 500-m spatial resolution. The new data from the improved Collection 6 MODIS Global Land Cover Type product were utilized in this work to increase and correct spatial resolution utilizing the EVI, LST, and NBAR measures [37].

### 2.3. Data Processing

The data products obtained from MODIS observations describe aspects of the land, seas, and atmosphere that may be utilized for local to global scale investigations of processes and trends. MODIS NDVI data from the MOD13Q1 product offer a Vegetation Index (VI) value per pixel. The Normalized Difference Vegetation Index (NDVI), also known as the Continuity Index to the existing NDVI, is generated by the National Oceanic and Atmospheric Administration-Advanced Very High-Resolution Radiometer (NOAA-AVHRR).

We first collected the available data of LULC and NDVI products of MODIS from the website. The analysis in this study was carried out over a period of two decades, commencing in 2001, and used LULC and NDVI data to measure changes in LULC and vegetation change typically over a period of roughly 5 years. MODIS LULC data were utilized from 2001 to 2020 based on availability, whereas MODIS NDVI data were used from 2001 to 2022. All the downloaded MODIS pictures were collected, preprocessed, mosaicked, projected, and processed using Arc GIS tools. MODIS13Q1 datasets with the same temporal resolution (16 days) and spatial resolution (250 m) were obtained from the MODIS website from 2000 to 2022. The time series data were checked for noise and parameter reconstruction to obtain correct parameter consistency in each pixel of the datasets for analysis. The previous land cover data of the study region were collected from the Ethiopian Geospatial Agency.

The processing stage was carried out using Arc GIS tools after getting the data from the website. The first stage was to ensure that the study's data covered the appropriate area. Mosaicking of separate pixels for each year of the dataset is the next step of the process. The layers were trimmed to the research region after mosaicking to a new raster dataset. Reclassifying the image was the next step of the analysis which helped to reduce the number of classes. There were 17 layers in the MODIS LULC image dataset. Among the 17 layers, we have only 10 layers available in the study region.  Table 2 shows the outcomes of marginal classes in the study region. The steps of mosaicking, projecting coordinating, and classifying were done for each year from 2001 to 2020. Mapping the LULC was the next step of the analysis (Figure 1).

The changes and dynamics of different land cover classes were investigated using net change analysis and cross-tabulating matrix analysis methods. Investigating the net change of various land cover categories and examining their patterns over time can provide some helpful fundamental information regarding land cover systems, while it is evident that it does not provide precise knowledge about the conversion of one class to another. In other words, determining which land cover class was changed to another or which class caused fundamental changes in other land cover classes is impossible [38].

### 2.4. Accuracy Assessment of LULC

The comparison between the classified image and the ground truth data can be verified by accuracy assessment. Accuracy assessment of a classified image is important for analyzing LULC changes and determining the acceptability of the classification process [39–41]. To check the accuracy of the LULC interpretation, 82 points were randomly selected in the study area and verified using Google Earth software. The initial step was to generate randomly generated points from the categorized LULC picture. For analytical assessment, these points are used as a reference between the categorized picture and the ground truth. The producer's accuracy (PA) (equation (3)), user's accuracy (UA) (equation (2)), overall accuracy (OA) (equation (1)), and kappa coefficient (*K*) (equation (4)) are the parameters used to evaluate the LULC classification. The equations for each assessment are presented as

(1) $$\begin{array}{c} \text{overall accuracy}=\frac{\text{total no}.\text{ of correct classified pixels}}{\text{No reference pixel}}\;*100, \end{array}$$

(2) $$\begin{array}{c} \text{user acuracy}=\frac{\text{no}.\text{ of correct classified pixels in each class}}{\text{Total no}.\text{ of classified pixels in that class}}*100, \end{array}$$

(3) $$\begin{array}{c} \text{procedure acuracy}=\frac{\text{no}.\text{ of correct classified pixels in each class}}{\text{Total no}.\text{ of referenced pixels in that class}}*100, \end{array}$$

(4) $$\begin{array}{c} \text{kappa coefficient }\left(T\right)=\frac{\left(\text{total sample}*\text{total corrected sample}\right)-\sum \left(\text{column total}*\text{ row total}\right)}{{\text{total sample}}^{2}-\sum \left(\text{column total}*\text{ row total}\right)}*100. \end{array}$$

### 2.5. Change Detection

The change detection is the process of finding differences in the state of an object by observing it at different points in time [21, 42]. The goal of change detection is to examine the variability in LULC recorded during a certain time period connected to a specific location. The general steps of the change detection model were as follows: data collection, preprocessing of remotely sensed data, image classification using appropriate logic, change detection using postclassification analysis, and vectorization of the raster change layers so that they could be manipulated in GIS tools. The change from one LULC class to another class between 2001 and 2020 was quantified. The distribution of the changes that occurred for the specified period is also presented in the matrix tables. A change matrix plots LULC changes from 2001 to 2020, assessing overall changes in LULC classes [4, 9]. The examination of alterations in LULC from 2001 to 2020 was also demonstrated through the measurement of variation, the amount of change, and the distribution of maps that highlight the spatial and temporal differences. To investigate the maximal range of change, spatial and temporal LULC alterations were detected for LULC types. Temporal changes were investigated using the total difference in areas for each LULC between two specific times, whilst spatial changes were investigated on a pixel-by-pixel scale to assess intraclass variations throughout these periods. The pattern of shifts was also exhibited to clarify the analysis of changes in LULC and NDVI.

The change in the area of each LULC identified in Arc GIS was calculated using Excel. The categorized picture is manipulated using Arc GIS software for the first phase of change detection analysis. Change detection analysis involves converting raster images into shapefiles, merging several layers, and calculating the area of each LULC. These steps were performed for each analysis period (2001, 2008, 2015, and 2020). The next step was to perform change detection using the intersection tool of Arc GIS.

## 3. Results

Arable land, evergreen broadleaf forest, deciduous broadleaf forest, grassland, permanent wetland, savanna, woody savanna, mixed forest, urban lands, and water bodies are the main LULC classes for the catchment (Table 2). Savanna and evergreen broadleaf forests are the main LULCs covering about 75% of the area. However, built-up areas and water bodies are the LULCs with the lowest area coverage (Figure 2).

### 3.1. Spatial Distribution of LULC and NDVI

The catchment is dominated by savanna grasslands as the main LULC type (Figure 2). Savana dominated most of the western plains of the catchment for the past two decades. Evergreen broadleaf forests are the other LULC type that represents the southeastern portions.

Distribution of the deciduous broadleaf forest is along the western portion of the evergreen broadleaf forest in the southeast. Cropland is distributed in an uneven and pitted pattern in the central part. Urban and built-up areas, mixed forests, and water bodies are the LULCs with the lowest area coverage. Forest areas are areas with higher elevations and the lowlands are dominated by savanna. The verdant regions exhibit a superior NDVI score of over 0.3, while the zones ranging from yellow to red depict lower NDVI values of under 0.3 (Figure 4). Typically, the western zone of the watershed encompasses a reduced NDVI value when compared to the southeastern forest areas, which boasts an NDVI value almost reaching 1.0.

### 3.2. LULC Change Detection

Here, we describe the changes between classes (conversion from one LULC class to another) and within classes. In this study, LULC change is detected as a change in LULC labels between 2001 and 2020 MODIS imagery. LULC change detection for the area is presented in the following tables and figures. The results are presented in a change matrix table that contains important information about the change from one LULC class to another class. The change matrix showing the land cover changes between 2001 and 2020 was generated from classified images of the respective periods and used to assess the overall changes in the LULC classes between 2001 and 2020.

#### 3.2.1. LULC Change (2001–2008)

**Figure 5:**
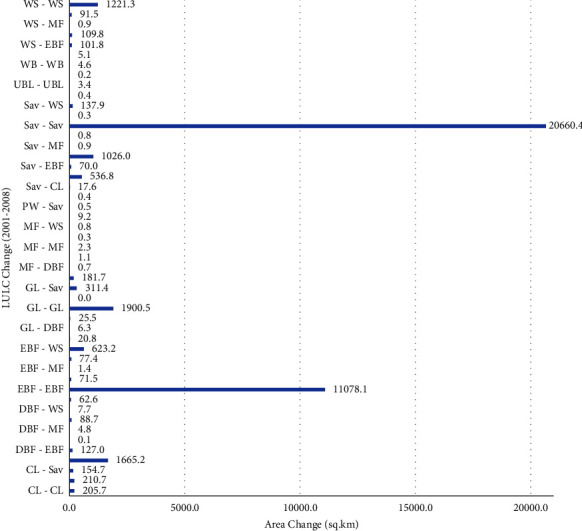
Change detection between 2001 and 2008.

The most important change between 2001 and 2008 was the change in large-scale savanna LULC to grassland (1026 km^2^), followed by the change from evergreen forest to woody savanna (623.2 km^2^) and grassland to savanna (311.4 km^2^) (Figure 5). In general, savanna, grassland, and cropland are the main LULCs where changes occurred in a shift to each other. The reduction and increment of each LULC in the given period are presented in Table 3.

#### 3.2.2. LULC Change (2008–2015)

**Figure 6:**
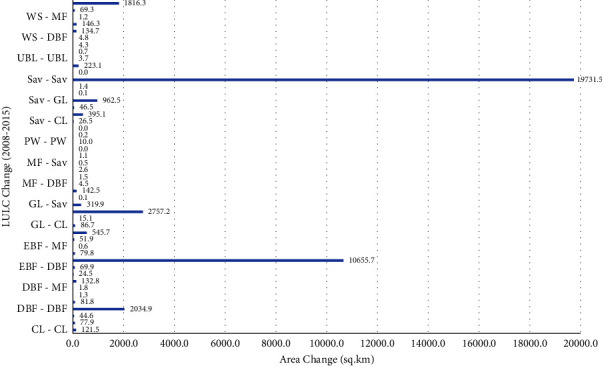
Change detection between 2008 and 2015.

The changes from large-scale savanna to grassland (962.5 km^2^) and from evergreen forest to woody savanna (545.7 km^2^) are the largest changes as in the previous trend (Figure 6). Savanna to deciduous forest (395.1 km^2^), grassland to savanna (319.9 km^2^), and savanna to woody savanna (223.1 km^2^) are the other major changes covering large areas. There are also significant changes such as woody savanna to grassland (146.3 km^2^), grassland to woody savanna (142.5 km^2^), woody savanna to evergreen forest (134.7 km^2^), and deciduous forest to savanna (132 km^2^). Table 3 shows a decrease of 1035.5 sq·km in the savanna area and a decrease of 468.7 sq·km in the evergreen forests. Grasslands and forested savannas were the areas with the greatest increase between 2008 and 2015.

#### 3.2.3. LULC Change (2015–2020)

**Figure 7:**
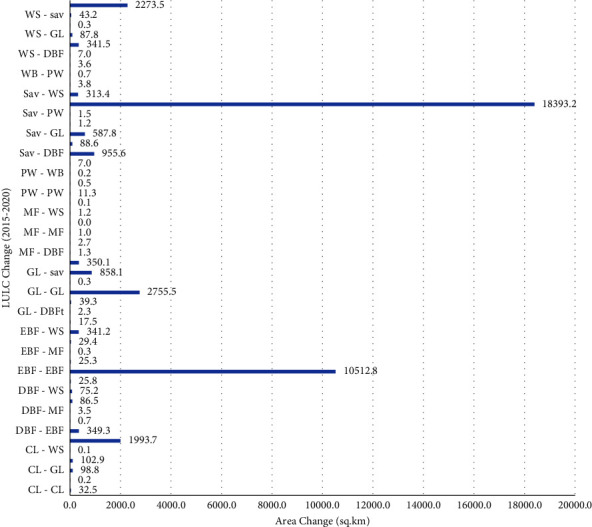
The change detection between 2015 and 2020.

Here, the switches from grassland to savanna (878.8 km^2^) and from savanna to grassland (587.8 km) are the most important changes. The change from the evergreen forest (350.1 km^2^), grassland (341.2 km^2^), and savanna (313.4 km^2^) to woody savanna is the main feature of the LULC change in this particular period (Figure 7). In addition, there was a change from woody savanna to evergreen forest (314.5 sq km). The change in coverage area between 2015 and 2020 is presented in Table 4. There have been major changes to the savannas and woody savannas LULC classes.

#### 3.2.4. LULC Change (2001–2020)

**Figure 8:**
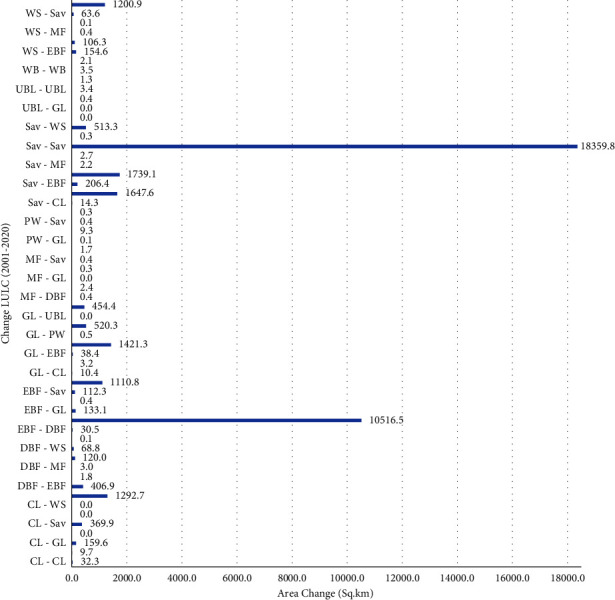
LULC change of the catchment (2001–2020).

The detection of changes revealed that the savanna has changed into extensive grassland (1739 km^2^) and deciduous forest (1640 km^2^) (Figure 8). The other major change is the change of EBF to a woody savanna (1110 km^2^). Savanna to woody savanna, grassland to woody savanna, deciduous forest to evergreen forest, cropland to savanna, and savanna to evergreen forest are the other major changes at 513, 454, 407, 369, and 206 square kilometers, respectively. The rate and percentage change between 2001 and 2020 are shown in Table 4).

**Figure 9:**
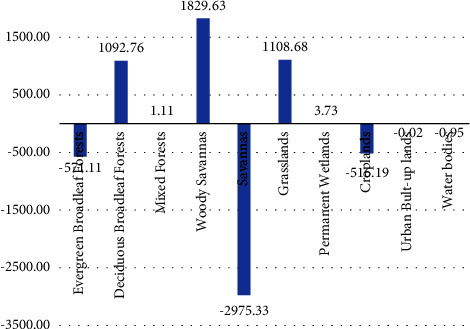
The changes in LULC between 2001 and 2020.

The changes in cover of the savanna, evergreen broadleaf forest, and croplands are the largest changes that occurred in the catchment, which decreased to −2975, −571, and −516 sq·km respectively, (Figures 9 and Table 4) between 2001 and 2020. In recent decades, there has been an increase in coverage for woody savannas, deciduous forests, and grasslands. There is no significant change for permanent wetlands, water bodies, and urban areas.

**Figure 10:**
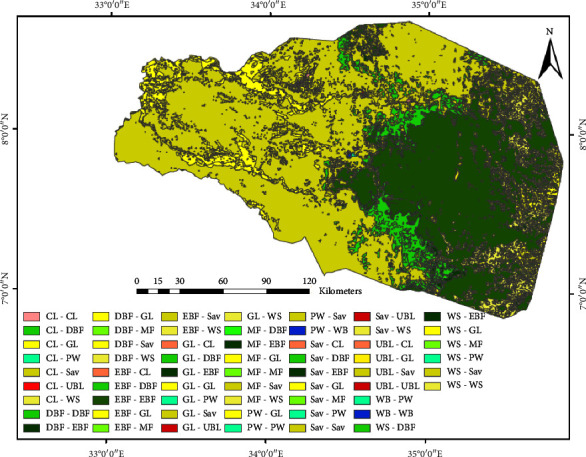
LULC change detection (2001–2020) map.

Savanna and forest are the major LULCs that cover a total of more than 70% of the area. Savannas are distributed in large areas in the west while forest dominated the southeastern part for the whole period of the analysis (2001 to 2020) (Figure 10). The increment grasslands in the savanna-dominated areas of the northwestern coroner give a new appearance to the LULC map. The sparse distribution of croplands in the savanna areas of the 2001 map also got a very tiny distribution in the 2020 map (Figure 2).

### 3.3. Trend of LULC (2001–2020)

**Figure 11:**
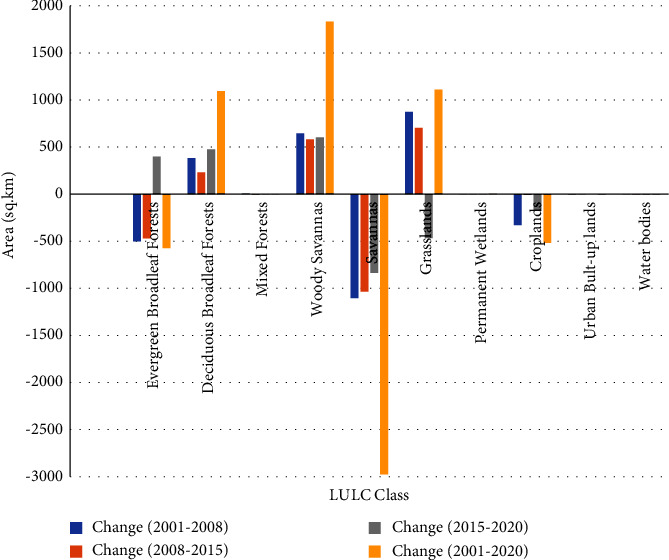
The trend of change in LULC between the four periods.

Uniform trends of increase, decrease, and uniformity (no change) are cases where trend detection results were displayed. In other words, no inconsistent status is seen in the trend analysis of the catchment. The LULC trend (Figure 11) shows that evergreen broadleaf forests, savannas, and cropland show a declining trend while deciduous broadleaf forests, woodland savannas, and grasslands are increasing from 2001 to 2020. There is no discernible trend in permanent wetlands, urban areas, and water bodies (Figure 11).

**Figure 12:**
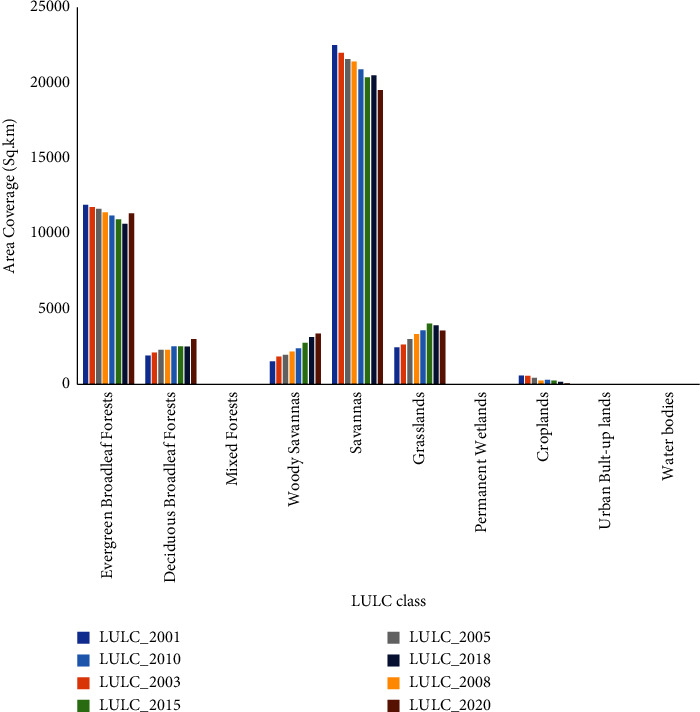
LULC trends between 2001 and 2020.

The changing trend analysis shows that perfect positive trends were seen for deciduous forests and woody savannas (Figures 11 and 12). Besides the change between 2015 and 2020, grassland shows a positive trend for most of the periods. For savannas and croplands, there was a negative trend in the past. With the exception of the most recent years (2015 and 2020), when they exhibit an upward trend, evergreen deciduous forests have historically shown a declining trend.

#### 3.3.1. Accuracy Assessment

An accuracy assessment was performed between the MODIS LULC data and Google Pro data from 82 random points taken from the study site. Accuracy is checked using the most commonly used assessment method called kappa accuracy. The matrix table (Table 5) displays the outcomes of precision verification by utilizing a randomly selected point from the LULC map and the Google Earth data. The details of the method and user accuracy for every LULC parameter are presented in Table 6.

#### 3.3.2. NDVI Characteristics

**Figure 13:**
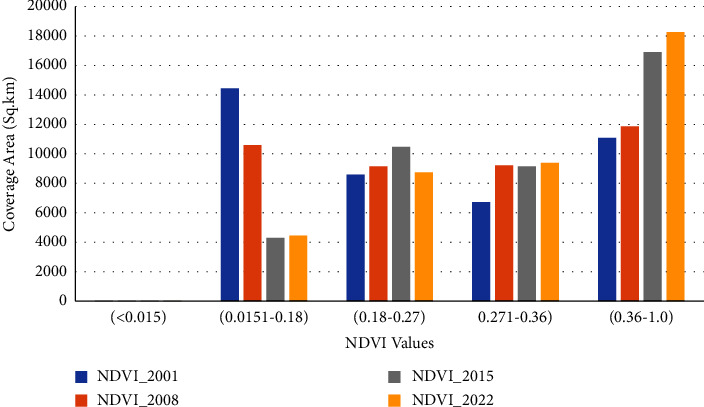
NDVI trends of the catchment (dry period).

**Figure 14:**
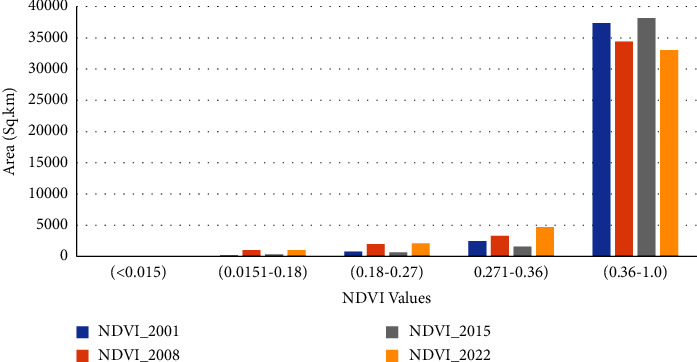
NDVI trends of the catchment (wet period).

The NDVI values are indices that indicate the health of vegetation in a specific area (Figure 3). The Vegetation Index value generally increased from 2001 to 2022. However, the increase in value is not uniform, and it is different in dry and wet period trends (Figures 13 and 14). In the dry period (January), it increased between 2001 and 2008, decreased between 2008 and 2015, and increased again between 2015 and 2022 (Figure 13). The wet period average value of NDVI shows an opposite trend to the dry period which decreased from 2001 to 2008, increased from 2008 to 2015, and again decreased from 2015 to 2022 with only a low range compared to dry period cases (Figure 14). The classified value of NDVI showed a similar general trend for dense vegetation area coverage. From 2001 to 2022, the vegetation cover and growth were improved by covering a large area. In addition to increasing the density of green vegetation in the southeastern part, the western corner areas were covered with green vegetation in late 2022 (Figure 3).

The dry period NDVI shows that dense vegetation coverage increased from 27% to 44% while barren land decreased from 35% to 10% between 2001 and 2020. During dry periods, a declining trend in the coverage of NDVI values (0.015 to 0.181) was observed between 2001 and 2022. On the other hand, during the same time period, the coverage area showed comparable growth for NDVI values greater than 0.27.

The dry period analysis of NDVI shows that the value range of the NDVI_2001 value (0.0151 to 0.18) covers the largest area followed by the value of the NDVI value (0.36 to 1.0) (Figure 13). In the wet period, the NDVI value of 2022 shows the largest value covered by the NDVI value (0.361–1.0) followed by the NDVI value (0.27–0.36). Generally, the greenness of vegetation increases from 2001 to 2022 (Figures 3, 13, and 14). The wet period analysis of NDVI shows that the majority of the area is covered by a value of 0.36 to 1.0 for all the four periods (Figure 14).

## 4. Discussion

Land-cover maps are critical variables in studying environmental dynamics. Change in LULC is unavoidable since it is a result of social and economic progress. However, it frequently comes with a high environmental cost. The expansion of agricultural land, for example, causes water pollution, sedimentation, erosion, and loss of biodiversity. Changes in land cover are usually caused by human activity such as urbanization, agricultural expansion, and deforestation, as well as natural occurrences such as wildfires, floods, and desertification [43]. Changes in land use and land cover (LULC) have recently been one of the most important and persistent causes generating changes in the Earth's land. This study articulates the LULC and NDVI changes for the last two decades using MODIS satellite images. The study discovered that LULC classes, namely, evergreen broadleaf forest, savanna, and water bodies, have reduced at the expense of natural vegetation covers such as shrubland and woodland. Change detection analysis was performed in order to understand the changes for the last period of time. Change detection analysis was performed in the Arc GIS environment to understand how a given area has changed between different time periods. We studied MODIS land cover categorization products from 2001 to 2020 and compared them to the produced MODIS NDVI trends to determine which land cover classes show trends and hence may be influenced by the change.

Net change analysis and cross-tabulating matrix analysis methods were used to analyze the changes and dynamics of various land cover classes. Investigating the spatial distribution maps of different land cover classes and establishing their proportion of the total area of the research, it was discovered that the savanna (47.8%) and evergreen broadleaf forests (27.7%) are the dominant LULCs encompassing around 75% of the area followed by grassland, woody savannas, and deciduous broadleaf forest with a coverage of 8.7% 8.2%, and 7.3%, respectively. However, cropland, permanent wetlands, mixed forests, built-up regions, and water have the least amount of coverage of the total area. The trend of LULC in the research region differs by LULC type from 2001 to 2020. Deciduous broadleaf forests, mixed forests, woody savannas, grasslands, and permanent wetlands all showed an increasing tendency. However, there was a declining trend in evergreen broadleaf forests, savannas, water bodies, and croplands.

The dynamics of transition from one land cover type to another were studied, revealing a reciprocal link between various LULC types. The rise in one LULC area was connected with a reduction in another LULC type. Decreased evergreen broadleaf forest, for example, is connected with increased deciduous broadleaf and mixed forest land. The decrease in savanna coverage may be related to an increase in grassland area. These reciprocal relationships were also observed in water bodies and permanent wetlands; a decrease in water body coverage may be associated with an increase in wetland regions.

In all four independent eras of investigation, the significant change in LULC was caused by a shift from the savanna to grassland. From 2001 to 2020, the transition from savanna to grasslands in each epoch diminishes the area covered. However, the reciprocal change in grassland to savanna LULC increased with a narrower range from 2001 to 2020 than the savanna to grassland shift. This indicates that large areas of savanna have changed to grassland nature of LULC over the past decades. The other major change was the change from evergreen broadleaf forest to woody savanna which increases from time to time. The findings also demonstrate that the evergreen forest of south-western Ethiopia changes its cover from time to time, posing a severe environmental risk to the region and the country as a whole.

In the research area, the greenness of vegetation increases with precipitation. The range of NDVI values for the dry period is reasonably spread. During the rainy season, however, most of the land turns green, with an NDVI value greater than 0.36. The greenness also follows the gradient of the slope. High-elevation places offer more lush green flora than gradual sloping lowlands.

The Vegetation Index results show that larger NDVI values occurred in forest and green vegetation areas, and the lowest value was found in barren and built-up uplands. The evergreen, deciduous forest, and mixed forest areas of the southeastern parts had NDVI values ranging from 0.30 to 1.0. Most of the savanna and woody savanna areas have NDVI values of 0.10–0.40. Wetlands and the water bodies have NDVI values of −0.05 to 0.05. Barren and built-up lands have NDVI values in the range of 0.050–0.14. In addition to increasing the density of green vegetation in the southeastern part, the western corner areas were covered with green vegetation in late 2022 (Figure 3). This change in NDVI corresponds to the change in LULC at the end of 2020 which shows an increment of grassland coverage in the western part of the study.

There are a number of similar studies that show comparative results of changes in LULC in Ethiopia as well as abroad. A study by Moisa et al. [33] on the Anger River Subbasin, western Ethiopia, shows results corresponding to our study. Other similar studies also have a corresponding result in western Ethiopia [43–45]. There are several studies on the database that have principles analogous to the present research. The work is similarly analogous to Mahmoudi et al.'s [38] study on detecting land cover changes in Baluchistan using the MODIS Land Cover product. Another comparable study was conducted by Somayajula et al. [46] on classification and validation of spatio-temporal changes in land use/land cover and land surface temperature of multitemporal images.

To manage overall environmental resources, an understanding of LULC variability in a given location is required. The suggested technique has numerous applications in land resource management. This sort of research provides firsthand knowledge regarding the surface covering. The LULC variability maps provide information for policymakers and general river basin planning in an area. The difference between this research and others is related to its areal coverage as well as its spatial and temporal resolution.

## 5. Conclusions

The present study shows how LULC and vegetation variability have changed significantly in western Ethiopia over the past two decades. This study evaluated and checked changes in LULC and NDVI patterns in western Ethiopia using MODIS satellite data from 2001 to 2020. Using MCD12Q1 and MOD13Q1 from 2001 to 2020 as data sources, this study examined the annual differences in LULC and NDVI for different years. The overall classification accuracy of LULC for the classified image from 2001 to 2020 was found to be 85.4% and the overall kappa statistics was 81.2%. The result of this study reveals that the major LULC in the catchment is savanna and forest. The cropland, savanna, and water bodies were reduced by 90%, 13%, and 19%, and woody savanna, deciduous forest, grassland, and permanent wetland increased by 119%, 52%, 45%, and 37%, respectively, over the past decades. The results also show that some water body areas have changed to permanent wetlands. The trend result showed that deciduous forests, woody savannas, and grasslands has increasing trends through 2001 to 2020. But the evergreen deciduous forest shows a decreasing trend for the given period. The most notable change was the change from savanna to other LULC classes such as grassland and forest. The result also showed that the NDVI value of the catchment was −0.046 to 0.98 in the driest month and it ranges from −0.093 to 0.99 for the wettest month. In the wettest month, 80% of the area has an NDVI value range of 0.36 to 1.0. This shows that 80 of the area is covered by green vegetation for the rainy season. The researcher concludes his research by stating that the catchment has achieved dual coverage of LULC and is losing that coverage that needs to be preserved for the future. Thus, this investigation foresees that the results could furnish insights into those responsible for overseeing water and land, as well as policymakers, in order to ensure the enduring management and growth of the area's natural assets.

## Figures and Tables

**Table 1:** Characteristics of MODIS LULC and NDVI data.

Short name, product name	MODIS NDVI	MODIS land cover type
Instrument	MOD13Q1	MCD12Q1
Processing level	Level-3	
Spatial resolution	250 m	500 m
Temporal resolution	16 days	Yearly
Availability	2001, present	2001, present

**Table 2:** Description of LULC of the catchment.

Symbol	LULC name	Description
EBF	Evergreen broadleaf forests	Dominated by evergreen broadleaf and palmate
DBF	Deciduous broadleaf forests	Dominated by deciduous broadleaf trees (canopy > 2 m)
MF	Mixed forests	Dominated by neither deciduous nor evergreen
WS	Woody Savannas	Tree covers 30–60% (canopy > 2 m)
Sav	Savannas	Tree covers 10–30% (canopy > 2 m)
GL	Grasslands	Dominated by herbaceous annuals (<2 m)
PW	Permanent wetlands	Permanently inundated lands with 30–60% water cover and >10% vegetated cover
CL	Croplands	At least 60% of area is cultivated cropland
UBL	Urban and built-up lands	At least 30% impervious surface area including building materials, asphalt, and vehicles
WB	Water bodies	At least 60% of area is covered by permanent water bodies

**Table 3:** LULC changes among 2001, 2008, and 2015.

LULC	LULC_2001		LULC_2008		LULC_2015		Change in 2001 to 2008		Change in 2008 to 2015	
	Area	%	Area	%	Area	%	Change	%	Change	%
EBF	11907.6	29.1	11405.7	27.9	10937.0	26.8	−501.8	−4.2	−468.7	−4.1
DBF	1893.9	4.6	2277.5	5.6	2509.6	6.1	383.6	20.3	232.1	10.2
MF	5.2	0.0	10.2	0.0	6.2	0.0	5.0	95.9	−4.0	−38.9
WS	1529.0	3.7	2173.9	5.3	2755.5	6.7	644.9	42.2	581.6	26.8
Sav	22495.8	55.0	21391.2	52.4	20355.8	49.8	−1104.6	−4.9	−1035.5	−4.8
GL	2452.4	6.0	3324.5	8.1	4027.7	9.9	872.0	35.6	703.3	21.2
PW	10.1	0.0	10.2	0.0	12.1	0.0	0.1	1.2	1.9	18.2
CL	573.2	1.4	244.2	0.6	235.4	0.6	−329.0	−57.4	−8.8	−3.6
UBL	3.8	0.0	3.7	0.0	3.8	0.0	−0.1	−2.5	0.1	2.0
WB	4.8	0.0	5.0	0.0	4.3	0.0	0.2	5.1	−0.7	−13.8

**Table 4:** LULC changes between 2001 and 2020.

LULC	2001		2015		2020		Change in 2015 to 2020		Change in 2001 to 2020		Rate of change	
	Area	%	Area	%	Area	%	Change	%	Change	%	km^2^/y	%
EBF	11907.6	29.1	10937.0	26.8	11336.5	27.8	399.4	3.7	−571.1	−4.8	−30.1	−0.3
DBF	1893.9	4.6	2509.6	6.1	2986.7	7.3	477.1	19.0	1092.8	57.7	57.5	3.0
MF	5.2	0.0	6.2	0.0	6.3	0.0	0.1	1.4	1.1	21.3	0.1	1.1
WS	1529.0	3.7	2755.5	6.7	3358.6	8.2	603.1	21.9	1829.6	119.7	96.3	6.3
Sav	22495.8	55.0	20355.8	49.8	19520.5	47.8	−835.2	−4.1	−2975.3	−13.2	−156.6	−0.7
GL	2452.4	6.0	4027.7	9.9	3561.1	8.7	−466.6	−11.6	1108.7	45.2	58.4	2.4
PW	10.1	0.0	12.1	0.0	13.8	0.0	1.7	14.4	3.7	36.9	0.2	1.9
CL	573.2	1.4	235.4	0.6	57.0	0.1	−178.4	−75.8	−516.2	−90.1	−27.2	−4.7
UBL	3.8	0.0	3.8	0.0	3.8	0.0	0.0	0.0	0.0	−0.6	0.0	0.0
WB	4.8	0.0	4.3	0.0	3.8	0.0	−0.5	−11.6	−0.9	−19.9	0.0	−1.0

**Table 5:** Categorized image (2020): accuracy assessment error matrix.

LULC	EBF	DBF	MF	WS	Sav	GL	PW	CL	UBL	WB	Total
EBF	9	1	0	0	0	0	0	0	0	0	10
DBF	1	9	0	0	0	0	0	0	0	0	10
MF	0	0	8	0	0	0	0	0	0	0	8
WS	0	0	1	8	1	0	0	0	0	0	10
Sav	0	0	0	0	16	2	0	0	0	0	18
GL	0	0	0	0	1	7	0	0	0	0	8
PW	0	0	0	0	0	0	3	0	0	2	5
CL	0	0	0	0	0	1	0	5	0	0	6
UBL	0	0	0	0	0	0	0	1	3	0	4
WB	0	0	0	0	0	0	1	0	0	2	3
Total	10	10	9	8	18	10	4	6	3	4	82

**Table 6:** Procedure and user accuracy assessment results of LULC 2020.

LULC class	Reference total	Classified total	Correct classified	Procedure accuracy	User accuracy
EBF	10	10	9	90.00	90.00
DBF	10	10	9	90.00	90.00
MF	9	8	8	88.89	100.00
WS	8	10	8	100.00	80.00
Sav	18	18	16	88.89	88.89
GL	10	8	7	70.00	87.50
PW	4	5	3	75.00	60.00
CL	6	6	5	83.33	83.33
UBL	3	4	3	100.00	75.00
WB	4	3	2	50.00	66.67

## Data Availability

Data used to support this study are available on request from the corresponding author.
